# A Decision Support System Coupling Fuzzy Logic and Probabilistic Graphical Approaches for the Agri-Food Industry: Prediction of Grape Berry Maturity

**DOI:** 10.1371/journal.pone.0134373

**Published:** 2015-07-31

**Authors:** Nathalie Perrot, Cédric Baudrit, Jean Marie Brousset, Philippe Abbal, Hervé Guillemin, Bruno Perret, Etienne Goulet, Laurence Guerin, Gérard Barbeau, Daniel Picque

**Affiliations:** 1 Institut National de la Recherche Agronomique, Unité Génie et Microbiologie des Procédés Alimentaires, Thiverval-Grignon, France; 2 Institut National de la Recherche Agronomique - Institut de Mécanique et d’Ingénierie, Talence, France; 3 Institut National de la Recherche Agronomique, Unité Sciences Pour l’Œnologie, Montpellier, France; 4 Institut Français de la Vigne et du Vin, Unité de VINs, Innovations, Itinéraires, TERroirs et Acteurs, Amboise, France; 5 Institut National de la Recherche Agronomique, Unité Vigne et Vin, Beaucouzé, France; 6 InterLoire, Tours, France; Jiangnan University, CHINA

## Abstract

Agri-food is one of the most important sectors of the industry and a major contributor to the global warming potential in Europe. Sustainability issues pose a huge challenge for this sector. In this context, a big issue is to be able to predict the multiscale dynamics of those systems using computing science. A robust predictive mathematical tool is implemented for this sector and applied to the wine industry being easily able to be generalized to other applications. Grape berry maturation relies on complex and coupled physicochemical and biochemical reactions which are climate dependent. Moreover one experiment represents one year and the climate variability could not be covered exclusively by the experiments. Consequently, harvest mostly relies on expert predictions. A big challenge for the wine industry is nevertheless to be able to anticipate the reactions for sustainability purposes. We propose to implement a decision support system so called FGRAPEDBN able to (1) capitalize the heterogeneous fragmented knowledge available including data and expertise and (2) predict the sugar (resp. the acidity) concentrations with a relevant RMSE of 7 g/l (resp. 0.44 g/l and 0.11 g/kg). FGRAPEDBN is based on a coupling between a probabilistic graphical approach and a fuzzy expert system.

## Nomenclature

Table 1 describes the name and the meaning of variables required in our study.

**Table 1:** Name and description of variables.

Name of the variable	Description	Unit
T	sum of average daily temperatures on a week	°C
RH	Mean ambient relative humidity on a week	%
Ins	Insolation duration on a week	h
Pl	Rainfall on a week	mm
Tmeanday	Mean temperature on a day	°C
Tmaxday	Maximal temperature by day	°C
RHday	Mean relative humidity of a day	%
Insday	Insolation duration on a day	h
Plday	Rainfall on a day	mm
S	Sugar concentration. Mean concentration measured on 200 grape berry	g/L
Ac	Total acidity. Mean concentration calculated on 200 grape berry	g/l Eq H2SO4

## Introduction

Agri-food is one of the most important sectors of the industry [1] and an important contributor to the global warming potential in the world. Sustainability issues pose a huge challenge for this sector. In this context, a big issue is to be able to predict the multiscale dynamics of those systems using computing science. Nevertheless mathematicians facing up to several bottlenecks: the variety of scales, the uncertainties, the out-of-equilibrium states, the complex quantitative and qualitative factors, the availability of data. We propose a methodology to implement robust predictive mathematical tools applied to this sector. More specifically it is applied to the wine industry but could be generalized to other agri-food applications. The wine industry involves a major part of SMEs (small and manufacturing enterprise) that should integrate innovation and are a strong support from regional and (inter-)national policies. The starting point of the quality and signature of the wine is the grape berry quality. Our study is focused on the prediction of the dynamics of the variables involved in the construction of this quality.

Grape berry quality depends on physiological and biochemical reactions taking place essentially from veraison to the harvest of grapes. Grape maturity is described by several variables, berry size, grape color, concentration of total soluble solids, acidity, phenolic compounds, anthocyanin contents. These variables guide the harvesting date which influence the quality of the wines [2,3,4,5]. Climate and weather conditions affect their evolutions [6,7].

In this context, it is relevant to propose decision support systems able to calculate reliable predictions of berry composition according to the meteorological conditions. Air temperature, rain fall, relative humidity, sunshine hours are well known to affect the grape ripening, mainly sugar concentration [8] and total acidity [7]. Anthocyanins level [9] are also important to predict but not measured by winegrowers inside the vineyard.

The decision of the optimal time to harvest mostly relies on the expert knowledge and the evaluation of the grape maturity [5]. In a context of optimization and sustainable “considerations” a big challenge for such an agri-food system is to propose robust mathematical predictive tools relying on knowledge integration [10]. Nevertheless it is not an easy task as regards to this specific domain. Indeed difficulties remain to develop and implement integrative mathematical tools for several reasons detailed in [11]. In the case of grape maturity, above the complexity of the reactions involved, several factors are to be emphasized: Data handling is time consuming and limited (one year for one experimental condition), available knowledge is fundamental to handle but expressed in different forms (equations, expert opinions, databases…), different formats (numeric, symbolic, linguistic…) and at different scales (microbiological, physicochemical, organoleptic…). To answer to this problematic, we propose, in the idea of coupling formalisms [12, 13], a decision support system combining a dynamic Bayesian network [14] with a fuzzy expert system [15] formalizing the available scientific and practitioners knowledge on the system.

DBNs are an extension of Bayesian networks (BNs) [16,17] that rely on the probabilistic graphical models where the network structure provides an intuitively appealing interface by which humans can model highly-interacting sets of variables and provides a qualitative representation of knowledge. Uncertainty pertaining to the system is taken into account by quantifying dependence between variables in the form of conditional probabilities using experimental data available [18].

Fuzzy logic is a convenient mathematical approach to cope with applications where expertise is present [19]. This theory is particularly well adapted for dealing with symbolic data manipulated by experts [11]. It has been successfully applied for decision support in vine applications essentially for two purposes. A first category is dedicated to unsupervised clustering approaches. For example, Urretavizcaya *et al*., [20] have implemented a fuzzy Cmeans for a precision viticulture purpose. Post-veraison information is used to define zones within the vineyard. A zoning procedure is achieved with criteria differentiating « top class » grape zones and standard ones. Tagarakis *et al*., [21] have used a fuzzy unsupervised method to delineate management zones using fuzzy clustering techniques and developing a simplified approach for the comparison of zone maps. Morari *et al*., [22] couple geo-electrical sensors and fuzzy clustering approaches to help in the delineation of zones upon the soil constitution.

A second category is about the development of fuzzy expert systems. For example, Fragoulis *et al*., [23] have developed a fuzzy expert system based on expert knowledge. It calculates an Environmental indicator Impact of Organic Viticulture and propose a decision support. Gil *et al*., [24] used a linear multiple regression and a fuzzy logic inference model to evaluate the effects of micrometeorological conditions on pesticide application for two spray qualities (fine and very fine). None of them propose a mathematical formalism able to exploit the two types of knowledge available in this domain: data and expertise and cope with their different type of uncertainties. An interesting study is proposed by Coulon *et al*., [25] that develop an expert model for environmental purposes. The aim is to predict the vine vigor level according to the most influential variables. It is based on a fuzzy expert system set up using data available, under restrictions proposed by experts. Nevertheless it is developed for classification purposes and not kinetic reconstruction and prediction.

We propose in this article to develop a decision support tool, based on new trends crossing over recent developments in computing science and food science. It is based on a coupling between a dynamic Bayesian network [14] and a fuzzy expert system [15]. The innovation and interest of the methodology is to be capable of sharing different sources of heterogeneous and fragmented information. It is done by the way of a coupling between mathematical approaches. Those approaches are selected upon the format of knowledge available and the advantage of each method. Dynamic Bayesian networks allow to represent and simulate complex stochastic dynamical systems. However, this formalism requires substantial knowledge to define the specific parameters (*i*.*e*. conditional probability distributions) which is a clear bottleneck in our domain. Experiments led along one year provide only one local climatic condition. In parallel, experts are capable of providing a macroscopic view of the system and expressing it by means of qualitative heuristics. Those experts have memorized in a symbolic way, the impact of different climatic conditions on the grape maturity along years of practice. It can not be directly used to set the conditional probabilities of the DBNs but can be easily handled using fuzzy logic. Once it is formalized in the form of fuzzy rules, the output can be easily projected on a numeric space using fuzzy membership functions. We thus propose to integrate inside DBNs the results of the simulations of the fuzzy expert system allowing a coupling between local and global knowledge. The paper is organized as follows. After a description of the material and methods, including a presentation of the mathematical concepts underlying the decision support tool, the decision support system so called FGRAPEDBN is described. Results and their analysis are presented in the next to finish by a conclusion and future works.

## Materials and Methods

### Experimental data

The study was conducted in 2006, 2007, 2008 and 2009 during the four or five weeks before the haverst on 28 parcels in different locations of Loire Valley (14 in Tours region and 14 in Angers region), the authorities IFV Tours and IFV Angers, Institut Français de la Vigne et du vin—Tours and Angers and the Chambre d’Agriculture d’Indre et Loire (represent the union of wine producers for the Loire Valley), gave us the necessary permissions and authorizations for each location. A whole of 456 points are treated including 4 or 5 points by kinetics for each parcel. During 2006, only the parcels of Tours region were included in the study. Temperature (°C), rainfall (mm) and relative humidity (%) were supplied by Meteo France meteorological stations located near and/or on the parcels. Solar radiation (in hours) was only given by one meteorological station located at Montreuil-Bellay, in the center of the area of study.

Each week, two lots of two-hundred berries of Cabernet Franc, with pedicels, were randomly picked up from each parcel at each ripening stage according to the method of Vine and Wine French Institute (ITV-France) [26] in order to limit the effects of the grape heterogeneity.

With a lot of two-hundred berries of each sampling, a crushing was realized with a blender, the must was then filtered through a Whatman paper filter. Reducing sugar concentration (g/l) was measured with a refractometer; total acidity (g/l Eq H_2_SO_4_) by the titration method.

### Knowledge handling

Knowledge has been formalized on the basis of a synthesis made by the scientists and the industry (Syndicats of Loire wine who have supported this study) in previous work and reports. Two types of experts were involved for this synthesis: 4 scientists and 5 winegrowers working on the two areas considered in this study.

### Models based on Gaussian process

Non parametric approaches relying on the Gaussian process such that Gaussian process latent variable, (swithching)-Gaussian process dynamic model are efficient tool for solving regression problem and are widely used in speech recognition, motion tracking *etc* where data are substantial and trajectories are well known [27, 28]. Assuming that an output Y follows a Gaussian process *GP(μ*,*R)*, the idea is to learn a mapping **y** = f(**x**) from a training sample {***X*,*Y***} = {**x**_i_,**y**_i_}_i = 1…N_ by maximizing the conditional probability:

$$Argmax_{\theta ,\beta }P\left(Y_{1},\ldots ,Y_{N}|X,\theta ,\beta \right)=Argmax_{\theta ,\beta }N(Y_{1},\ldots ,Y_{N}|\mu_{\beta }\left(X\right),R_{\theta }\left(X,X\right))$$ (1)

where *N*(***Y***|*μ*_*β*_(***X***),*R*_*θ*_(***X***,***X***)) is a multivariate Gaussian distribution with a mean function *μ*_*β*_(***X***) which has to be defined according to the available knowledge (*e*.*g*. linear, non linear function, moving average …) and a covariance matrix *R*_*θ*_(***X***,***X***) whose entries my be given by the kernel function:

$$R_{\theta }\left(x_{i},x_{j}\right)=\theta_{1}e^{-\theta_{2}|\left|x_{i}-x_{j}\right|{|}^{2}}$$ (2)

Once the parameters learnt, given a new observation x*, the prediction of y* is estimated by means of the distribution [27]

$$p\left(y^{*}|x^{*},Y,X,\theta ,\beta \right)=N\left(\mu \left(x^{*}\right),\sigma^{2}\left(x^{*}\right)\right)$$ (3)

where

$$\mu \left(x^{*}\right)=\mu_{\beta }\left(X\right)+{\left[Y-\mu_{\beta }\left(X\right)\right]}^{t}R_{\theta }{\left(X,X\right)}^{-1}R_{\theta }\left(x^{*},X\right)$$ (4)

$$\sigma^{2}\left(x^{*}\right)=R_{\theta }\left(x^{*},x^{*}\right)-R_{\theta }{\left(x^{*},X\right)}^{t}R_{\theta }{\left(X,X\right)}^{-1}R_{\theta }\left(x^{*},X\right)$$ (5)

This approach may be extended for dynamic model by puting Y = [y_2_,…,y_N_] and X = [y_1_,…,y_N-1_].

### Dynamic Bayesian networks

A Bayesian Network [16,17] is a graph-based model of a joint multivariate probability distribution that captures properties of conditional independence between variables. On one hand, it is a graphical representation of the joint probability distribution and on the other hand, it encodes independences between variables. Formally, a Bayesian network is a directed acyclic graph (DAG) whose nodes represent variables, and whose missing arcs encode conditional independences between the variables. This graph is called the structure of the network and the nodes containing probabilistic information are called the parameters of the network. Dynamic Bayesian networks (DBNs) are an extension of Bayesian networks [14] in which nodes ***X***(*t*) = (*X*_*1*_(*t*),…,*X*_*n*_(*t*)), representing discrete random variables, are indexed by time *t* and provide a compact representation of the joint probability distribution *P* for a finite time interval [1,τ]. That means that, the joint probability distribution *P* may be written as the product of the local probability distribution of each node and its parents as follows:

$$\;P\left(X\left(1\right),\cdots ,X\left(\tau \right)\right)=\prod_{i=1}^{n}\prod_{\text{t}=1}^{\tau }P(X_{i}\left(t\right)|U_{i}\left(t\right)\text{)}$$ (6)

where *U*_*i*_(.) denotes the set of parents of a node *X*_*i*_(.) and *P*(*X*_*i*_(.)|*U*_*i*_(.)) denotes the conditional probability function associated with the random variable *X*_*i*_(.) given *U*_*i*_(.). *X*_*i*_(*t*) is called a “slice” and represents the set of all variables indexed by the same time *t*. This factorization of the joint probability distribution, based on graphical information, facilitates the representation and use of large models. It represents the beliefs about possible trajectories of the dynamic process. DBNs assume the first-order Markov property which means that the parents of a variable in time slice t must occur in either slice *t*-1 or *t*. Moreover, the conditional probabilities are time-invariant (*first order homogeneous Markov property*) meaning that *P*(*X*(*t*)|*U*(*t*)) = *P*(*X*(2)|*U*(2)) for all *t* in [1, σ]. Hence to specify a DBN, we need to define the intra-slice topology (within a time slice), the inter-slice topology (between two time slices), as well as the parameters (i.e. conditional probability functions) for the first two time slices. The structure of a model can be explicitly built on the basis of knowledge available in the literature and parameters can be automatically learned without a priori knowledge on the basis of a dataset (known as parameter learning).

The techniques for learning DBNs are generally extensions of the techniques for learning BNs. Different methods exist to learn about the structure or the parameters from substantial and/or incomplete data [29, 30]. In our work, the topology of graph is obtained from scientific knowledge and the most commonly used and simplest method to estimate parameters consist in compute the occurrence rate in the training data.

The use of such DBNs consists in ‘‘query” expressed as conditional probabilities. The most common task we wish to solve is to estimate the marginal probabilities known as Bayesian inference:

$$P(X\left(t\right)|O\left(t^{\prime }\right)=o\left(t^{\prime }\right),\;\forall \;t\prime \;\epsilon \left[1,\tau \right])$$ (7)

where ***X*** is a set of query variables, and *O* is a set of evidence variables (for example, in food processing, *X* might be the variables representing the physicochemical properties of a product and *O* might be the variables representing the observed environmental conditions). In general, DBN inference is performed using recursive operators and Bayes’ theorem (given a way of calculating *P*(***X***(*t*)|*O*(*t'*)) from the knowledge of *P*(***X***(*t'*)|*O*(*t*)), [14]) that update the belief state of the DBN as new observations become available [14].

### Fuzzy logic theory

Fuzzy logic was proposed by Zadeh in 1965 [31]. It is an extension of the set theory by the replacement of the characteristic function of a set by a membership function whose values range from 0 to 1. Soft transitions between sets are thus obtained and allow the representation of gradual concepts as well as the representation and the inference of linguistic rules stemming from expertise. It is particularly adapted for taking human linguistic and reasoning processing into account [32, 33]. Fuzzy models can be written in an easy form to understand linguistic rules. Those rules link at a symbolic level the inputs to the outputs of a physical system [11]. For example a rule like: “a high mean day temperature combined with other factors potentially increases the sugar concentration in a grape berry” can be processed by such a system. Similarly, an essential fuzzy notion is the fuzzy membership function. A fuzzy set E in universe of discourse U can be defined by Eq 8:

$$\begin{array}{l} \text{E}=\left\{\left(\text{u,}\mu_{\text{E}}\text{(u)}\right)\backslash u\in \text{U}\right\}\\ \mu_{\text{E}}\text{:U}\to \left[\text{0,1}\right] \end{array}$$ (8)

**Fig 1:**
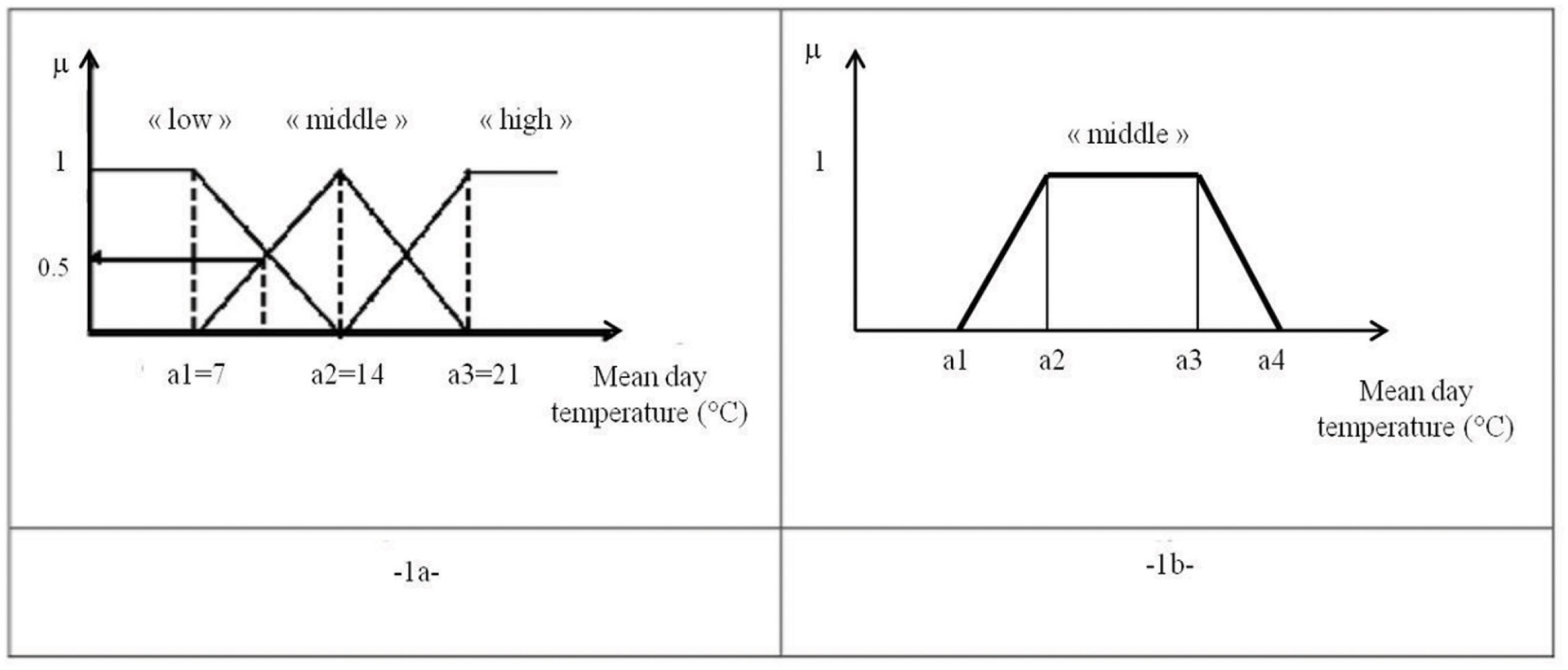
Fuzzy linguistic functions. -1a- example of the linguistic variable of a mean day temperature defined on triangular functions; -1b-An example of a trapezoidal function for the symbol “middle”.

*μ*_*E*_ is thus the membership function of set E. It represents the set of membership grades *μ*_*E*_(*u*) of a numerical variable u mapped to a fuzzy set E. It allows the linking of real numerical variable to a given linguistic variable. The value of the membership grade is a real number within the interval [0,^1^]. For example Fig 1a represents a projection of the mean day temperature measurements in °C versus the linguistic term quantifying the impact of it as regards to the grape maturity through symbols “low”, “middle” and “high”.

This notion gives the way to link a numeric variable to a linguistic variable often manipulated by the operators. In fact fuzzy memberships are used to describe how much an object belongs to a linguistic notion. Going back to Fig 1a, a mean day temperature of 10.5°C belongs to “low” with a membership degree of 0.5 and to “middle” with a membership degree of 0.5. It means that its impact on the maturation will be mitigated.

$$\mu \text{(x)}=\left\{ \begin{array}{l} \text{0}\begin{array}{cc} & \text{(x}<{\text{a}}_{\text{1}}\text{)} \end{array}\\ \frac{\text{x}-{\text{a}}_{\text{1}}}{{\text{a}}_{\text{2}}-{\text{a}}_{\text{1}}}\begin{array}{ccc} & {\text{(a}}_{\text{1}}\leq \text{x}<{\text{a}}_{\text{2}}\text{)} & \end{array}\\ \frac{{\text{a}}_{\text{3}}-\text{x}}{{\text{a}}_{\text{3}}-{\text{a}}_{\text{2}}}\begin{array}{cc} & {\text{(a}}_{\text{2}}\leq \text{x}<{\text{a}}_{\text{3}}\text{)} \end{array}\\ \text{0}\begin{array}{cc} & {\text{(a}}_{\text{3}}\leq \text{x)} \end{array} \end{array}\right.$$ (9)

Membership functions can be expressed through various representations. The representations most widely used are triangular (Eq 9) for a given triplet series of parameters a_1_, a_2_, a_3_, represented in Fig 1a for the mean day temperature quantification. Trapezoidal functions using four parameters are also regularly used defined then with 4 parameters a_1_ to a_4_ (see Fig 1b). Rules are computed using the direct application of Zadeh’s compositional rule of inference presented in Perrot and Baudrit [34]. Triangular norms and conorms manipulated in this model are respectively the bounded sum and the product. An activated grade $\alpha_{R_{j}}$ is calculated for each rule *R*_*j*_ of the knowledge basis using this compositional rule. Suppose for example rules *R*_*j*_, *j* = 1 to n with n the total number of rules, involving 2 variables A and B (for example A can be the mean day temperature in °C and B the day rainfall level in mm). Each variable is associated to a linguistic notion *i*_1_ (for example “high”) for A and *i*_2_ for B (for example “low”). ($\mu_{i_{1}}(A)$) noted $a_{i_{1}}$ and ($\mu_{i_{2}}(B)$) noted $b_{i_{2}}$ are the membership degrees to those symbols. The activated grade for a rule *R*_*j*_ involving *i*_1_ and *i*_2_ for A and B (for example if A is *i*_1_ and B is *i*_2_ then the class is *C*_*jk*_ for the rule *j* and the output *k*), is T($a_{i_{1}}$,$\;b_{i_{2}}$) with T the triangular conorm. $\alpha_{R_{j}}$ is then equal to $a_{i_{1}}\times b_{i_{2}}$ for a product selected as Tconorm. Each rule *R*_*j*_ is associated by the experts to a class for each output *k* (for example a class of impact on the total sugar concentration upon a given mean day temperature and a given day rainfall level). The equation applied to calculate the resulting impact Pclass for each output C_*k*_ (pclass) for *k* = 1…m crossing over all the rules is then (Eq 10):

$$\text{Pclass}(C_{k})=\frac{\underset{j}{\sum }\alpha_{R_{j}}\times P_{jk}}{\underset{j}{\sum }\alpha_{R_{j}}}$$ (10)

where *P*_*jk*_ is the conclusion of the rule *j* for the class *C*_*k*_ and *k* = 1 to m, m equal to 2 in our paper (sugar concentration and total acidity).

### Integrate fuzzy logic inside Dynamic Bayesian network

Assume that *X*_*i*_(*t*) are all categorical variables and let $\theta_{ijk}^{t}$ be the probability that *X*_*i*_(*t*) = *x*_*k*_, given that its parents *U*_i_(*t*) have possible values *x*_*j*_ (corresponding itself to a vector where *j* represents the vector of parents of *i*), i.e.

$$\theta_{ijk}^{t}=P\left(X_{i}\left(t\right)=x_{k}|U_{i}\left(t\right)=x_{j}\right)\;\text{for}\;\{\begin{array}{c} i=1,\ldots ,n\\ j=1,\ldots ,c_{i}\\ k=1,\ldots ,r_{i} \end{array}$$ (11)

where *r*_*i*_ is the number of values that node *i* can take and *c*_*i*_ is the number of distinct configurations of *U*_*i*_(*t*). As DBNs assume the first-order homogeneous Markov property (*i*.*e*. *P*(*X*_*i*_(*t*+1) = *x*_*k*_|*U*_*i*_(*t*+1) = *x*_*j*_) = *P*(*X*_*i*_(*t*) = *x*_*k*_|*U*_*i*_(*t*) = *x*_*j*_) leading to $\theta_{ijk}^{t}\;=\;\theta_{ijk}$ for all *t*∈[1,*τ*]. The used method to estimate and update DBN parameters $\theta_{ij}\;=\;(\theta_{ij1},\ldots ,\theta_{ijr_{i}})$ consists in using the conjugate prior multinomial probability distributions known as Dirichlet distributions *θ_ij_* ∼ Dir($\alpha_{ij1},\ldots ,\alpha_{ijr_{i}}$) [29,30]. If we have an available experimental database in which event (*X*_*i*_(*t*) = *x*_*k*_|*U*_*i*_(*t*) = *x*_*j*_) occurs *N*_*ijk*_ times, the posterior variable (*θ*_*ij*_|database) then follows a Dirichlet distribution (*θ*_*ij*_|database) ~Dir(${N_{ij1}+\alpha }_{ij1},\ldots ,{N_{ijr_{i}}+\alpha }_{ijr_{i}}$) and the expected a posteriori gives as estimation:

$$\theta_{ijk}=\frac{N_{ijk}+\alpha_{ijk}}{\sum_{\text{k}=1}^{{\text{r}}_{\text{i}}}N_{ijk}+\alpha_{ijk}}$$ (12)

where *α*_*ijk*_ = 1/*r*_*i*_ inducing an uniform prior distribution over *θ*_*ij*_ allowing to take into account the lack of data. Parameters may be then updated with a simulated database stemming from the results of the previous fuzzy model simulation, *i*.*e* (see [18]):

$$\theta_{ijk}=\frac{N_{ijk}^{S}+\alpha_{ijk}^{\prime }}{\sum_{\text{k}=1}^{{\text{r}}_{\text{i}}}N_{ijk}+\alpha \prime_{ijk}}$$ (13)

where $\alpha_{ijk}^{'}$ corresponds to the previous sum of *N*_*ijk*_+*α*_*ijk*_ and $N_{ijk}^{S}$ corresponds to the number of occurrences inside simulated database.

### Validation of the decision support system

A cross-validation methodology is achieved to validate the decision support system. The validation of the model is based on a 10-fold cross-validation [35]. The set of all parcels for the four vintages from 2006 to 2009 has been randomly partitioned into ten equal size subsamples. From the ten subsamples, a subsample is retained as the validation data for testing the model, and the remaining nine subsamples are used for the parameter learning of DBN. This processing is then repeated ten times.

Validation of the model is achieved using the RMSE (Root Mean Square Error calculus), Eq 14. and the correlation coefficient R^2^, Eq 15.

$$RMSE(X)=\sqrt{\frac{1}{N}\sum_{i=1}^{N}(x_{i}^{\text{mod}el}-x_{i}^{\text{exp}eriment})²}$$ (14)

$$R²=1-\frac{\sum_{x_{i}\in X}(x_{i}^{\text{mod}el}-x_{i}^{\text{exp}eriment})²}{\sum_{x_{i}\in X}(\bar{X}-x_{i}^{\text{exp}eriment})²}$$ (15)

The maximal error of prediction for the sugar concentration is fixed at 8.5 g/L by experts, equivalent to an error of 7.5% on the total possible variation (126 to 139 g/L). It is indeed directly linked to the alcoholic degree of the wine legally controlled (8.5 g/L is equivalent to 0.5 alcoholic degree). The acceptable error is also fixed by experts to 7.5% of the maximum scale deviation for the others outputs. It is equivalent to 0.41g/L for the acid concentration.

### The decision support system for grape berry maturity prediction

Two models are developed and coupled to integrate the maximum of knowledge available. A first expert model so called FGRAPE formalized the expert memory of what happens during the ten past years and its consequences on the grape berry maturity kinetics. It is then coupled to a dynamic Bayesian network (DBN) expressing the dynamic of the system on the basis of conditional laws extracted from the data basis representing 4 years of climatic conditions. DBN parameters are updated using the results of simulation of FGRAPE. It is achieved using Eqs 12 and 13 presented above. It leads to the decision support tool proposed in this paper and so called FGRAPEDBN.

### Gaussian and DBN model

The modelling has been done for the two physico-chemical indicators of maturation namely sugar, total acidity measured every week by the winegrowers. The retained environmental variables are temperature (T), sun exposure (Ins), relative humidity (RH), pluviometry (Pl). The aim is to develop a mathematical model capable of describing the behavior of sugar, total acidity concentration over the maturation step regarding environmental conditions according to available knowledge.

In the formalism of Gaussian process, we assume that the couple (Ac_t+1_, S_t+1_) ~ GP(μ_β_(*X*_t_),R_β_(*X*_t_,*X*_t_)) where X = (Ac_t_,S_t_,T_t_,Ins_t_,RH_t_,Pl_t_). According to Eq 1 and available data, the objective is to maximize the conditional probability

$$Argmax_{\theta ,\beta }P\left(Y|X,\theta ,\beta \right)=\;Argmax_{\theta ,\beta }N\left(Y_{1}|\mu_{\beta_{1}}\left(X\right),R_{\theta }\left(X,X\right)\right)\times N\left(Y_{2}|\mu_{\beta_{2}}\left(X\right),R_{\theta }\left(X,X\right)\right)$$ (16)

where ***Y***_**1**_ = [Ac_2_,.., Ac_N_], ***Y***_**2**_ = [S_2_,.., S_N_], and ***X*** is a 6×N-1 matrix. Mean functions *μ*_*β*_(***X***) will be estimated from training data [m_t_,Act,S_t_,T_t_,Ins_t_,RH_t_,Pl_t_] where m_t_ corresponds to a moving average over ***Y***. (*i*.*e* m_t_ has a form equal to m_t_ = 1/N×Σ_k_ Y_t-k_).

**Fig 2:**
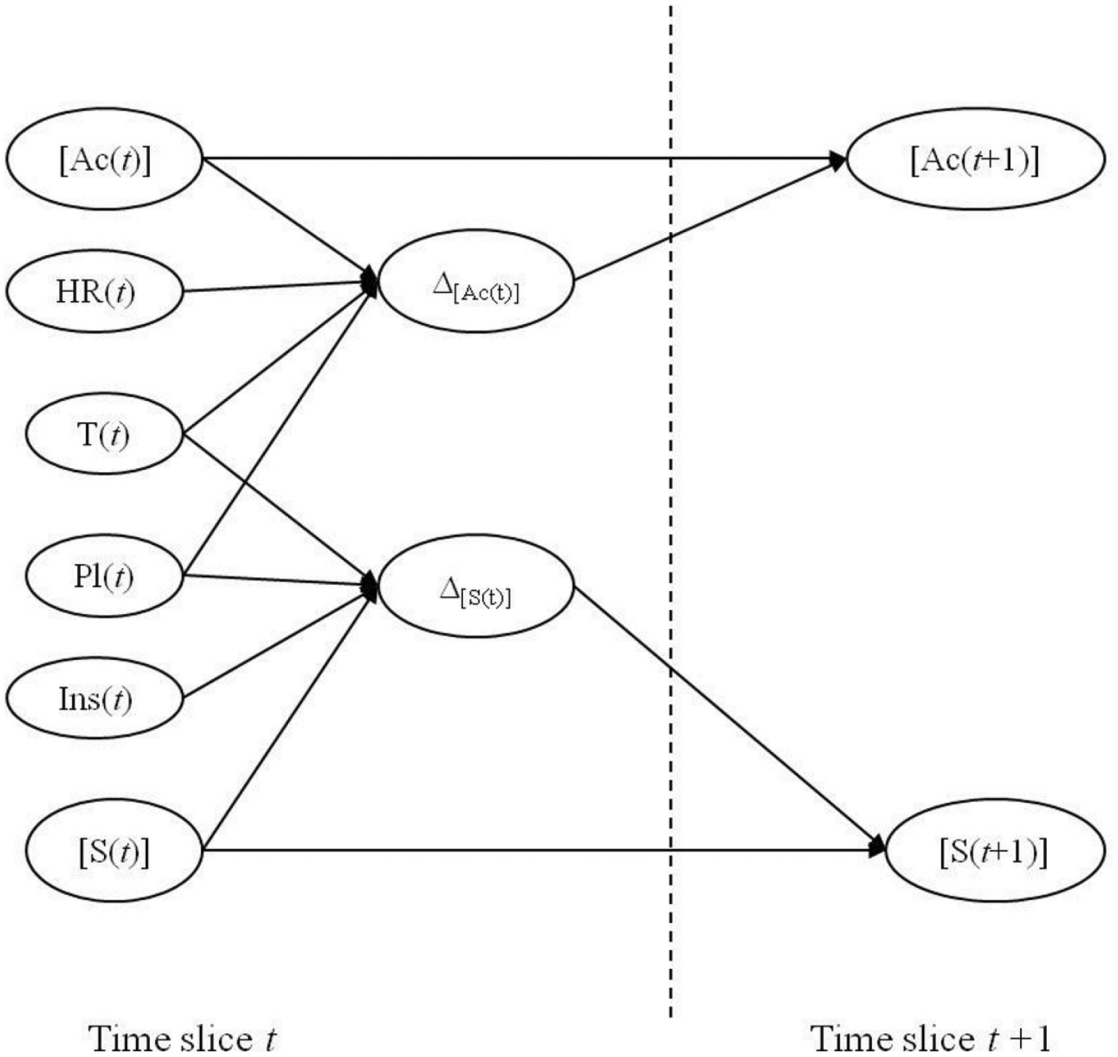
Dynamic Bayesian network representing the coupled dynamics of maturity indicators [Ac] (total acidity) and [S] (sugar) influenced by environmental climatic conditions RH, T, Pl and Ins Δ denotes variation.

Regarding the formalism of DBN Fig 2 displays the structure of the model making it possible to represent the coupled dynamics of maturity indicators [Ac] and [S] influenced by environmental climatic conditions HR, T, Pl and Ins. Table 2 displays the ranges of values of each variables.

**Table 2:** Lower and upper bounds of variables.

Variable	Bounds
T (F)	[60, 175]
Ac (g/L)	[2.9, 8.4]
Ins (h)	[0,80]
HR (%)	[50, 100]
Pl (mm)	[0,100]
S (g/L)	[106, 237]
Δ_S_ (g/L) by week	[–14, 36]
Δ_Ac_ (g/L) by week	[-2.2, 0.4]

### FGRAPE

**Fig 3:**
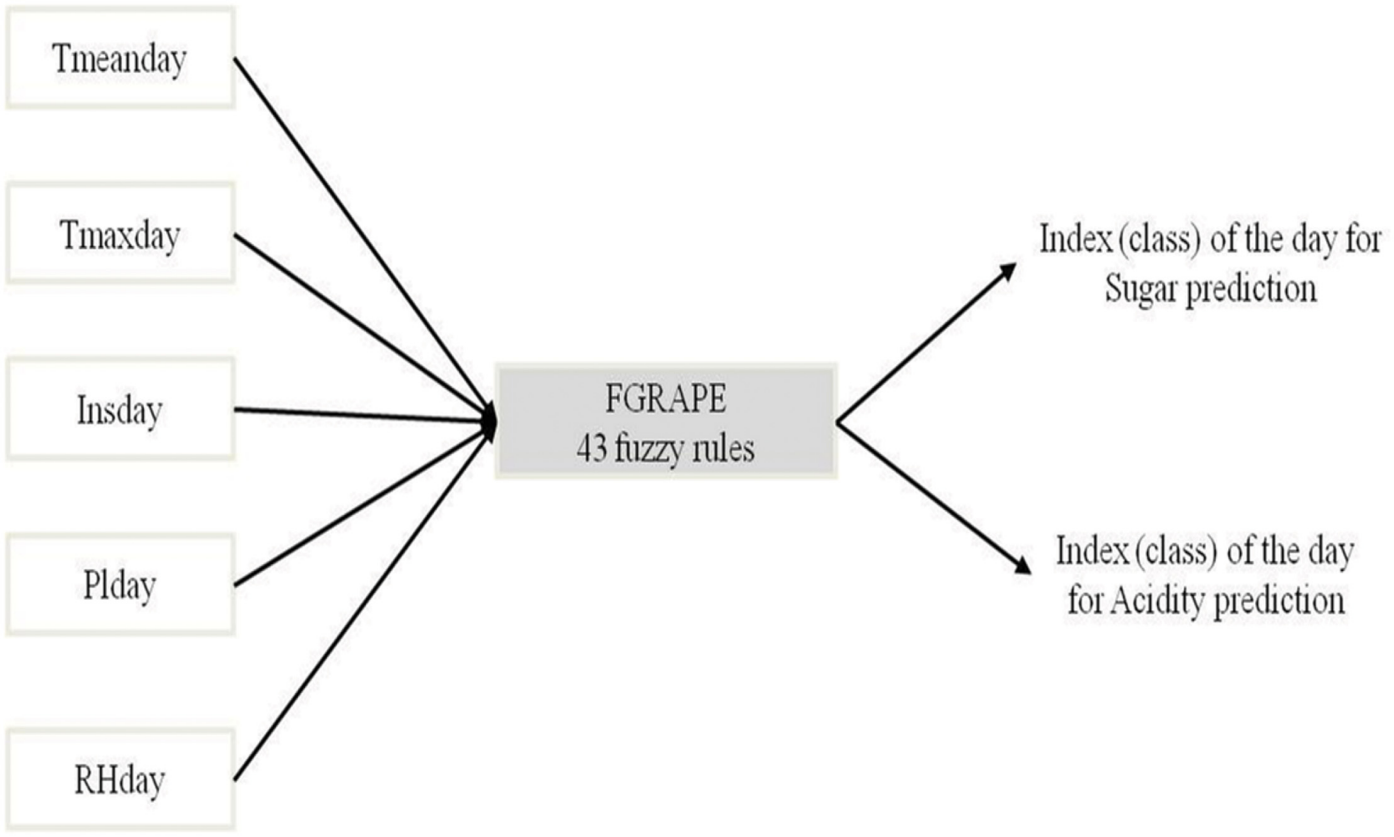
Inputs/outputs of FGRAPE.

FGRAPE represents the technological knowledge about the macroscopic behavior of the grape wine memorized by the experts during their years of practice. It is only built on what they have observed and measured: climate, sugar mean concentration (S) and total acidity (Ac). Fuzzy logic is used for expert knowledge computation. Fig 3 displays the inputs/outputs of the system. The output is expresses in terms of four indexes of day for sugar and acidity predictions, so a total of 8 indexes for the two outputs predicted upon the climatic conditions.

Table 3 presents the experts explanation of those indexes. It involves five inputs combinations: Tmeanday, Tmaxday, Insday, RHday and Plday. Table 4 illustrates combinations driving the outputs towards an index of day equal to 2. The parameters defining the fuzzy membership functions of each input are presented Table 5. 43 fuzzy rules are defined aggregating the 3×3×3×2×4 = 216 possibilities. For example a day with a Tmeanday middle, a Tmaxday low, Plday low and Insday low, whatever RHday takes a value of 1 for the index while a day with a Tmeanday middle, a Tmaxday low, Plday low, Insday middle and a low RHday takes a value of 2. An example of composition is detailed in Table 4.

**Table 3:** Description of the indexes related to day climatic conditions for the two outputs of FGRAPE: sugar concentration and total acidity.

Index levels	Sugar evolution per day (g/L)	Description for the sugar	Acidity evolution per day (g/L)	Description for the acidity
0	-1	Bad climatic conditions. Generally a high pluviometry with a dilution effect in the berries	+0.3	Bad climatic conditions for the maturation. Not enough water
1	+1	Not favorable climatic conditions. Limiting conditions for one input or a combination of inputs.	0	Not favorable climatic conditions. Limiting conditions for one input or a combination of inputs.
2	+3	Standard day for the region.	-0.2	Standard day for the region.
3	+5	Exceptional day for the grape maturity. High increase of sugar. It can be induced by a concentration phenomenon	-0.4	Exceptional day for the grape maturity. High consumption of malic acid or dilution phenomenon

**Table 4:** Example extract for 6 rules of FGRAPE driving towards a day index of a value 2.

Rule	Tmeanday	Tmaxday	Insday	Plday	RHday
1	middle	low	middle	low	low
2	middle	low	high	low	Low or middle or high
3	middle	low	middle	middle	low
4	middle	low	high	middle	Low or middle or high
5	middle	middle	middle	low	Low or middle or high
6	middle	middle	high	low	middle

**Table 5:** Fuzzy membership parameters of FGRAPE.

Variable	Linguistic variables	Parameters
Tmeanday	« low »; « middle »; « high »	(-∞, 7, 13]; [7, 13, 15, 21]; [15, 21, +∞)
Tmaxday	« low »; « middle »; « high »	(-∞, 26, 30]; [26, 30, 32, 35]; [32, 35, +∞)
Insday	« low »; « middle »; « high »	(-∞, 4.5, 5.5]; [4.5, 5.5, 7.5, 8.5]; [7.5, 8.5, +∞)
RHday	« low »; « high »	(-∞, 70]; [85, +∞)
Plday	« low »; « middleminus »; « middleplus », « high »	(-∞, 4, 6]; [4, 6, 13, 17], [13, 17, 25, 35]; [25, 35, +∞)

The resulting output calculated for a week by FGRAPE is computed Eq 17.

$$Output_{i}(week_{t})=Output_{i}(week_{t-1})+k\times \sum_{j=1,7on\Delta t}Index_{outputi}FGRAPE(day)$$ (17)

Where $\sum_{j=1,7on\Delta t}Index_{outputi}FGRAPE(day)$ is the sum on the 7 days contained in the week t of the values of the index predicted day by day by the fuzzy rules of FGRAPE for each output i (sugar or acidity) and k is a constant adjustment parameter fixed for each year based on expert criteria about soil considerations and global climatic impacts (equal to 0.8 for 2006, 1 for 2007, 0.9 for 2008 and 0.7 for 2009).

### A coupling between DBN and fuzzy logic: FGRAPEDBN

DBNs are very useful when few things are known about the phenomena of system but they need substantial database to estimate parameters. In our application, this database is really hard to acquire (1 year, 1 kinetic) which is generally the case in our domain. Fuzzy logic is then used to translate another source of knowledge, the expert knowledge expressed in the form of qualitative heuristics, into a data basis directly usable by the DBN. FGRAPEDBN represents the scientific knowledge about the principal conditional links that can be established between the grape maturity indexes studied and the climate conditions.

**Fig 4:**
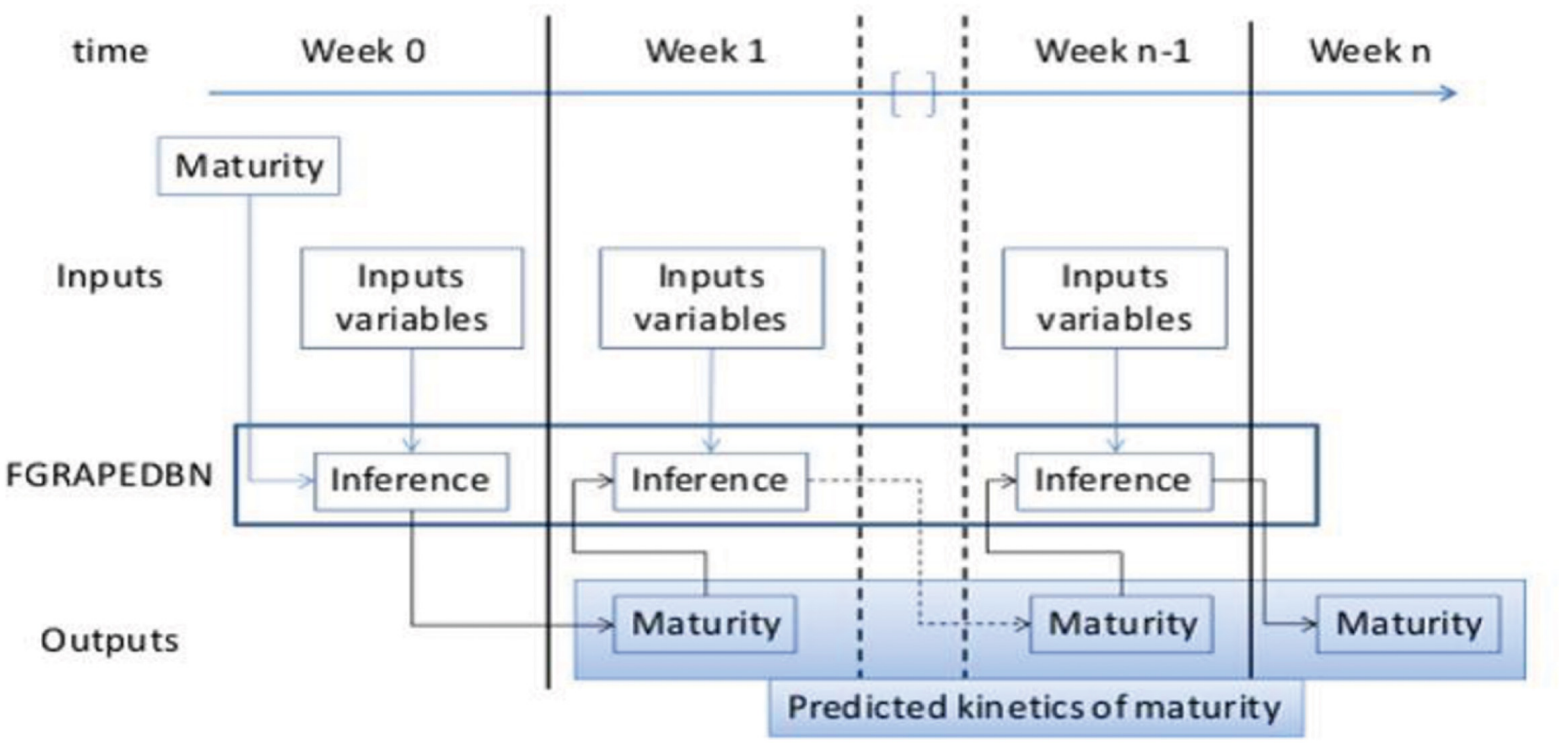
diagram of FGRAPDBN prediction.

The prediction of the decision support tool starts by an initialization of S and Ac on the basis of measurements achieved during the first week of the maturation, followed by simulations week by week of the dynamic Bayesian network based on predictions of the outputs for the week-1 (see Fig 4).

**Fig 5:**
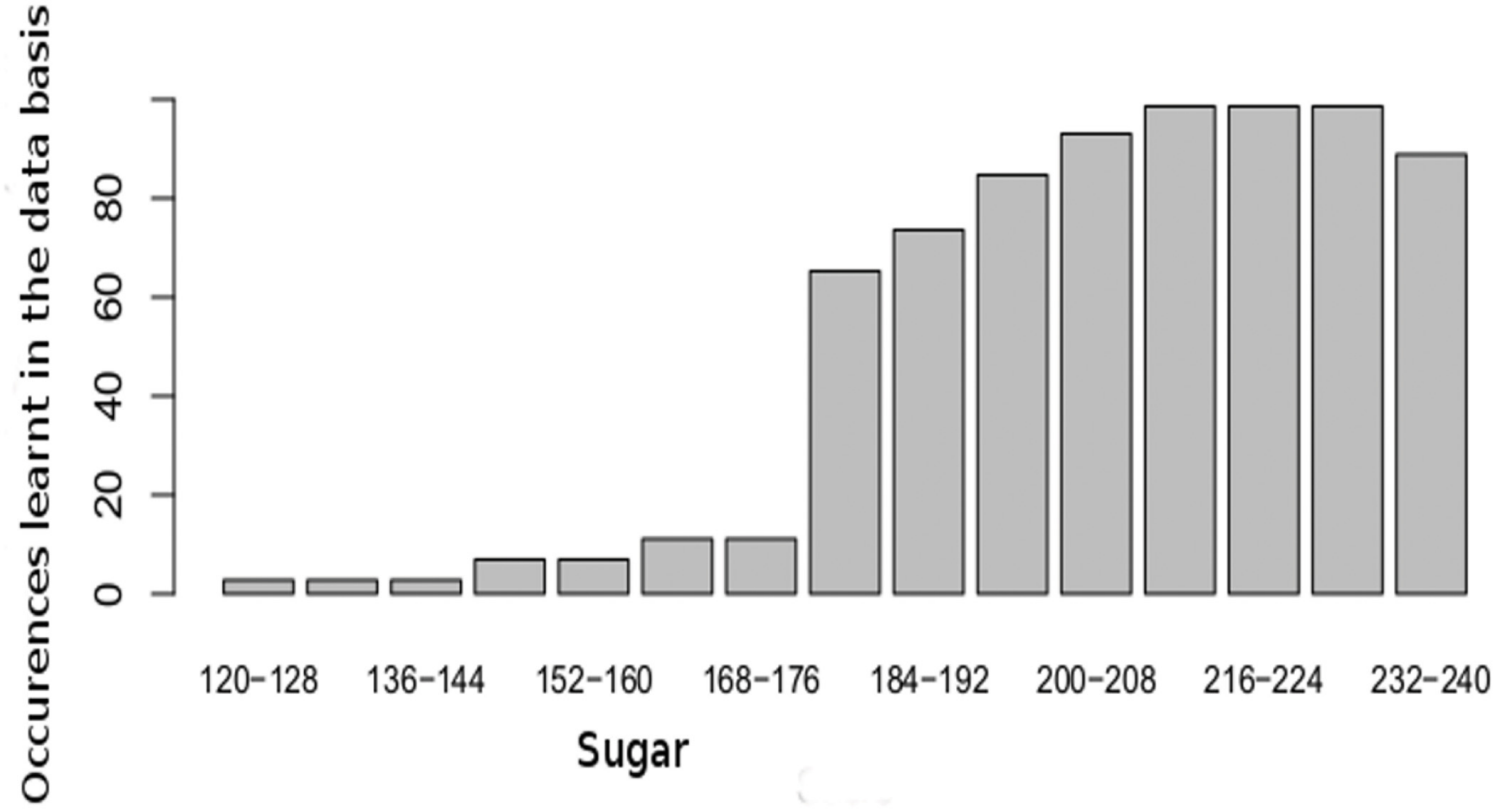
Occurrences of sugar found in the data basis (4 years) and learnt by the DBN over the whole duration of the maturation experiments.

On the basis of the conditional probabilities learnt using the data basis presented in Eq 12, FGRAPE is used to update the parameters of the DBN model upon a methodology proposed in Eq 13. For example Fig 5 displays the occurrences learnt by the DBN for the sugar concentration. If around 80 samples lead to a sugar concentration of 184–192 g/L, far less samples have been learnt for lower sugar concentrations on the four years of observations included in the data basis. The aim is the enrichment of the observations by a parameter upgrading of DBN using FGRAPE. The final purpose is to propose a robust decision support tool able to cover a large spectrum of climate conditions.

**Fig 6:**
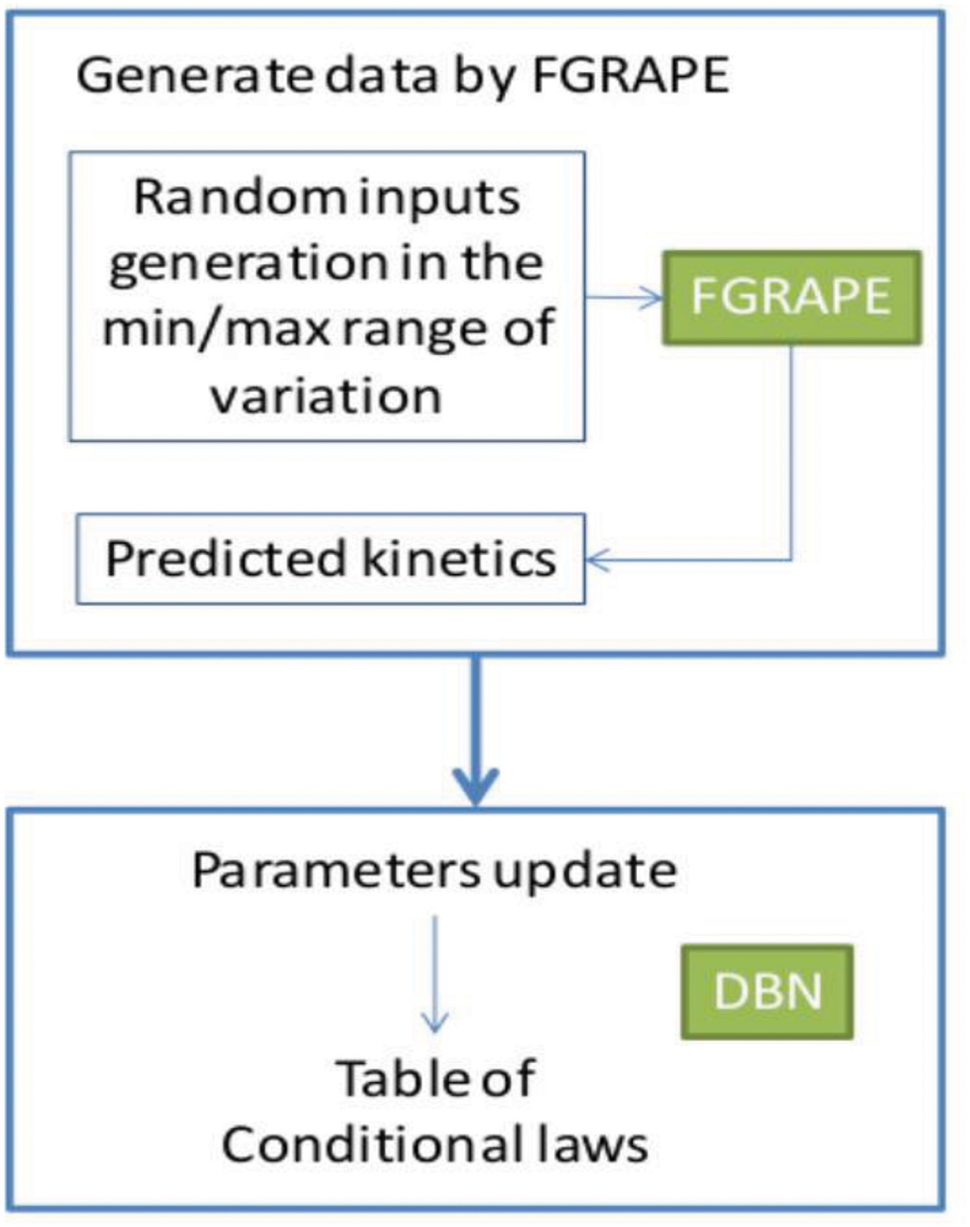
Model coupling diagram.

**Fig 7:**
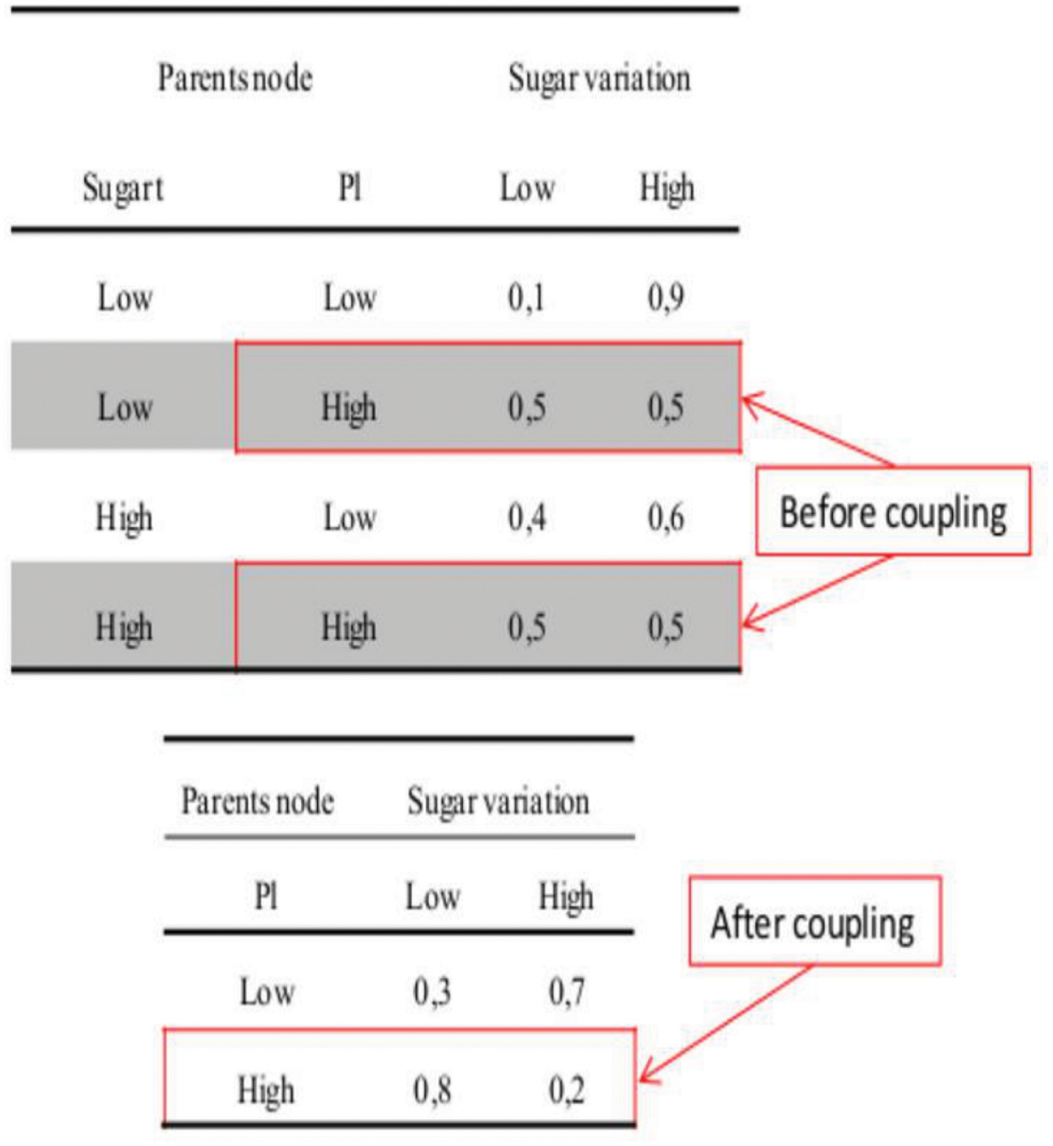
An example of knowledge integration through parameters updating using FGRAPE predictions. An equiprobability for high conditions of rainfall has been replaced by a higher probability for low concentration of sugar when rainfall is high (0.8 low and 0.2 high replace 0.5 low and 0.5 high before update).

100 different random configurations are generated and predictions of FGRAPE are used to upgrade the DBN parameters (Fig 6). Climate conditions for one day are approximated on the basis of the conditions measured for one week divided by 7. Tmaxday is also estimated by adding an aleatory increment to Tmean selected in the range [Tmean, Tmax] on the selected week. An example of upgrade is presented in Fig 7 where equiprobability is upgraded by results proposed by FGRAPE. It is for conditions never encountered in the data basis (sugar(*t*-1) low or high and Pl high).

## Results

### DBN predictions

The aim is to test the representative and predictive character of the model. The mean value $\overset{-}{\;X}$ has been chosen as the post-processing in order to predict final results, *i*.*e*:

$$\bar{X}\left(t\right)=\sum_{x}xP(X\left(t\right)=x|\left\{O\left(t^{\prime }\right)=o\left(t^{\prime }\right),\;\forall \;t^{\prime }\in \left[1,\tau \right]\right\})$$ (18)

where *P*(*X*(*t*) = *x*|{*O*(*t*') = *o*(*t*'),∀*t*'∈[1,*τ*]}) are marginal probabilities, *t* is on the order of week, *X* is total acidity (*resp*.sugar) and *Ac*(1),*S*(1) are initial concentrations;, *O*(*t*) = {*HR*(*t*),*Pl*(*t*),*Ins*(*t*),*T*(*t*)}, *t*∈[1,*τ*] are observed environmental conditions from time 1 to τ. All DBN parameters are initialized and updated by means of Eq 12, from an experimental database containing the monitoring of maturation from 2006 to 2009 on 26 parcels. Model simulations may then be compared to sugar and total acidity concentrations measured inside berry grapes over the maturation period for different parcels and different vintages.

The validation of model is based on a 10-fold cross-validation. A good root mean square error (see Table 6) is obtained for total acidity and sugar concentration that shows the accuracy of the model.

**Table 6:** Root mean square error (RMSE) relative to variables total acidity and sugar concentrations measuring the differences between values predicted by the dynamic Bayesian network (DBN), Gaussian process model (GP) and the raw measurements.

Error Measure	Sugar (g/L)	Total acidity (g/L)
RMSE (DBN)	7,9 g/L	0,48 g/L
RMSE (GP)	10 g/L	1 g/L

In order to compare our approach with the results of the Gaussian process model, we have estimated the hyper parameters (*β*,*θ*) according to Eq 16 leading to obtain unsatisfying predictions (see RMSE (GP) in Table 6). The inaccuracy of gaussian process model may stem from several reasons:

1. the assumption of the normality of studied processes,
2. the choice of the covariance matrix *R*,
3. the objective function is non-convex being able to lead to local minima in Eq 16

The formalism of Dynamic Bayesian Networks permits to relax these constraints.

### FGRAPE predictions

**Fig 8:**
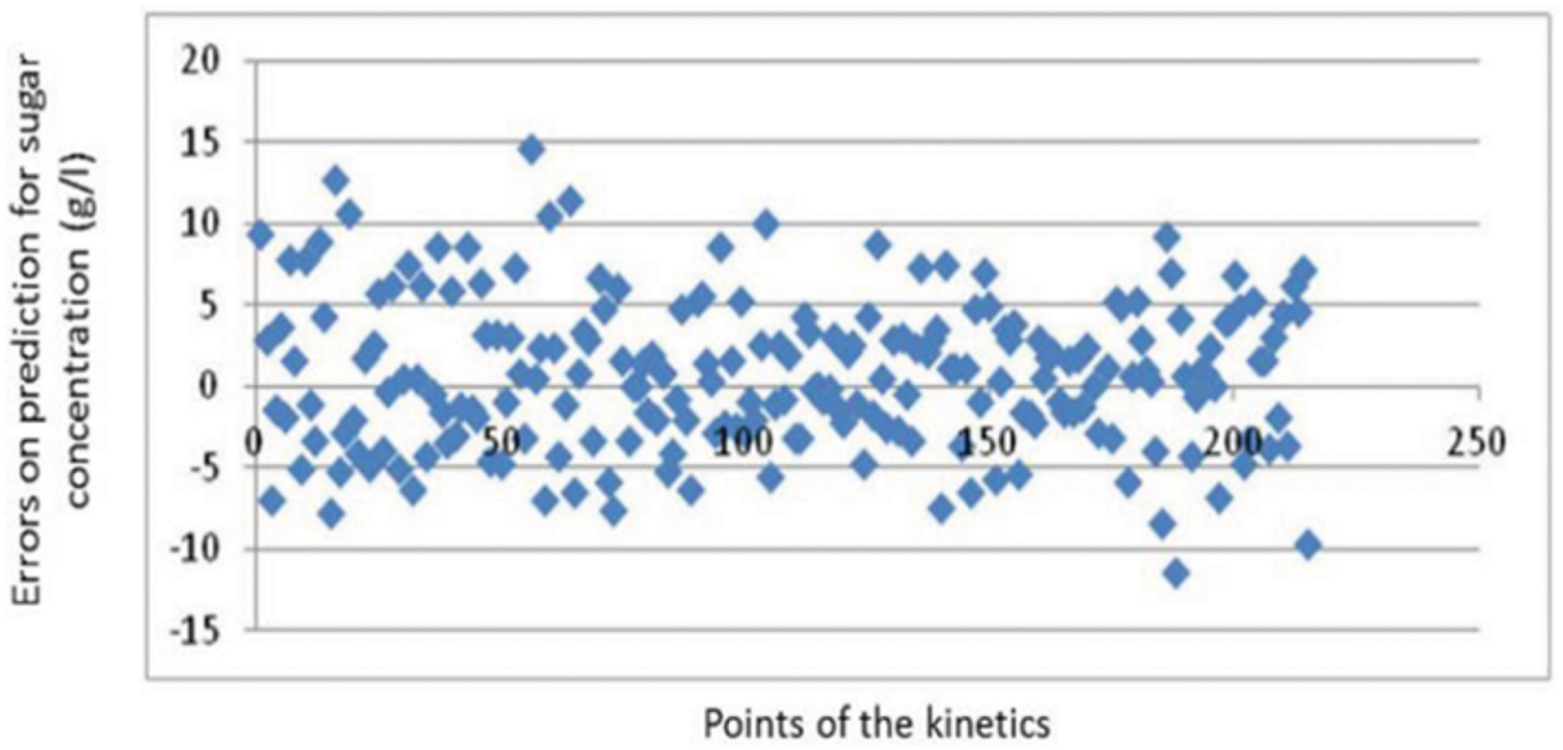
Scatter plot of the errors of prediction of FGRAPE for the data of the Tours appellation.

Before coupling the two models, the relevance of FGRAPE was tested. Table 7 presents the results of simulations and Fig 8 an example of errors for the sugar predictions on one appellation area.

**Table 7:** Results of prediction of FGRAPE.

Error Measure	Sugar (g/L)	Total Acidity (g/L)
R^2^	0.88	0.74
RMSE	6.11	0.55

It shows a good relevance of the expert rules computation applied on the years studied in the data basis even if RMSE for those specific years are slightly above the limit fixed for Ac predictions (cf. cross validation section). It can be explained by a generic knowledge covering a large spectrum of climatic conditions (R2 relatively good) with a counterpart of a loss in accuracy for specific conditions (see for example points for the Tour region in Fig 8 with errors greater than 8.5 g/l). It nevertheless validates the approach. Moreover this macroscopic model can be generalized to broader climate variations than those registered in the data basis. For these reasons, it has been used to complete the conditional laws set up in FGRAPEDBN only based on the memory of four years.

### Decision support tool FGRAPEDBN predictions

Predictions of FGRAPEDBN are presented in Table 8. Results of simulation are in good adequacy with the observations, with a RMSE below or near the sensitivity threshold fixed for sugar and acidity prediction, respectively 8.5 g/L and 0.41 g/L.

**Table 8:** decision help tool FGRAPEDBN: results of validation.

Error measure	Sugar (g/L)	Total Acidity (g/L)
Maximal deviation	148–239	2,9–7,5
RMSE	7,0	0,44
R^2^	0,82	0,77

**Fig 9:**
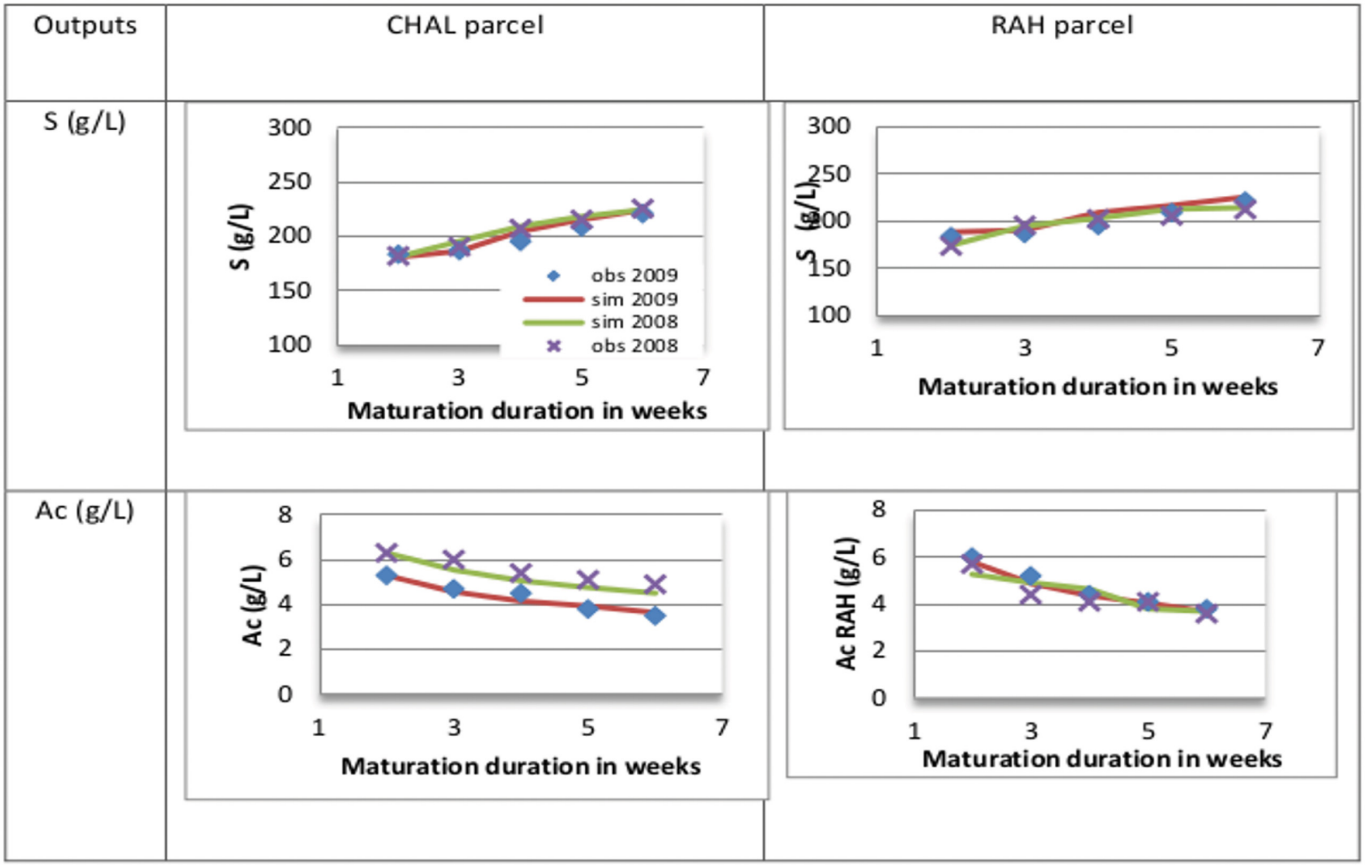
Prediction of FGRAPEDBN for two parcels CHAL and RAH during the whole maturation duration for two years 2008 and 2009 for sugar and Ac.

Results are in accordance with the reality. It can be depicted in Fig 9 for four dynamics of S and Ac on 2 areas and two years and in Table 6 for the global results of validation. Indeed a good prediction is observed for the experimental kinetics all along the weeks. Quantitative errors are very low for the sugar with more significant ones for Ac, for example on the third week for RAH. It is also interesting to notice the difference of dynamics according to the different years for a same area and the different dynamics for two areas during the same year which are globally well reproduced trough the FGRAPEDBN predictions. For example in 2009 the Ac evolution in the area CHAL starts at 5.3 g/L by comparison to an Ac for the RAH area which starts at 6 g/L. Moreover the slope in the two first weeks is divided by two for the CHAL parcel.

## Discussion

**Fig 10:**
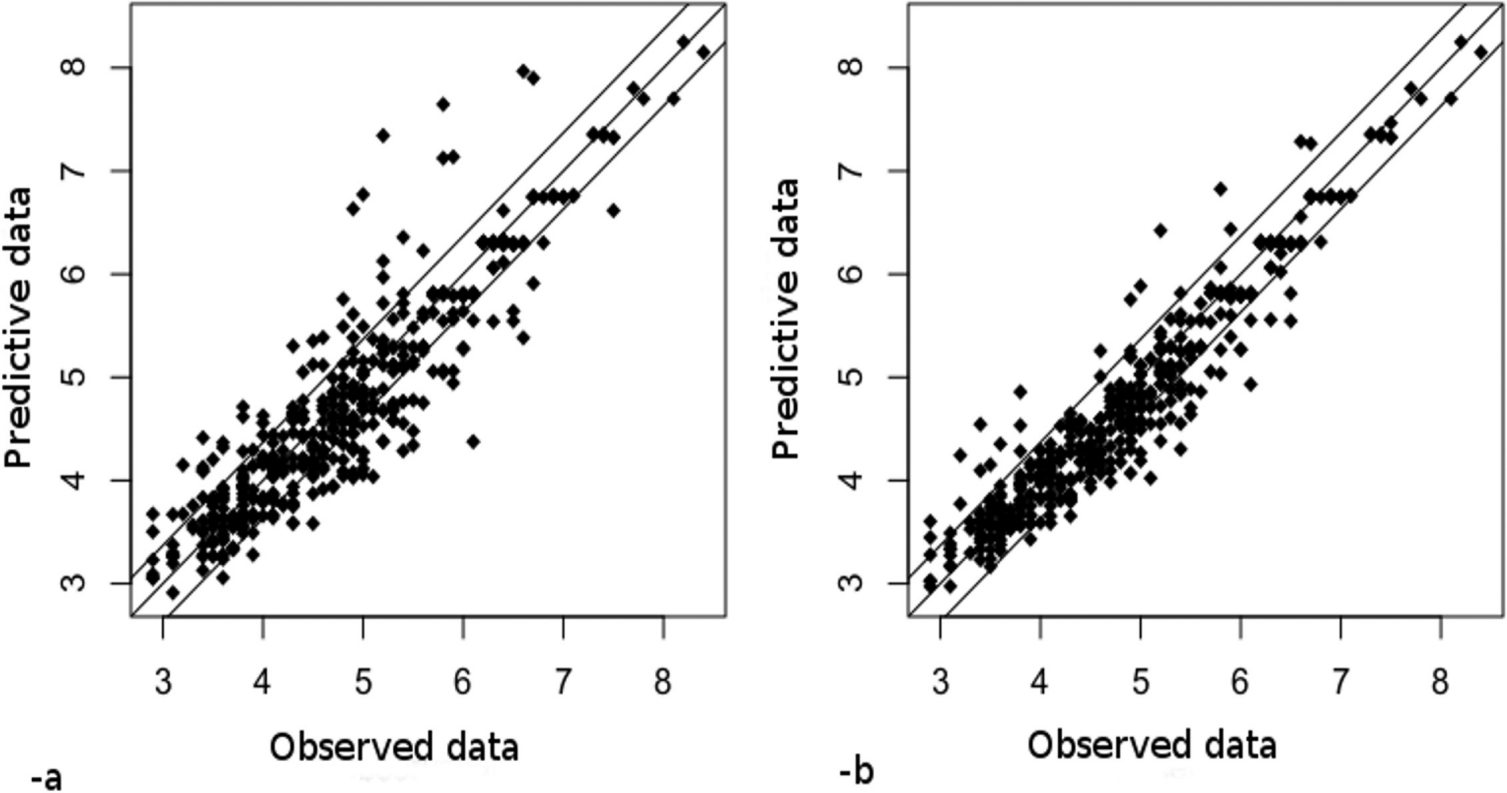
Scatter plot of simulations versus experiments for the maturation predictions before coupling—a-and after—b-for Ac.

Fig 10 depicts the value added of our approach of knowledge integration as regards to the results reached for example for the total acid prediction. After coupling, results are globally more correlated to the experiments with a scatter plot more compact around the correlation line. The RMSE for Sugar predictions, is 7.9g/l before coupling (prediction of the DBN alone) and 7g/l after coupling. It means that the coupling between the FGRAPE model and the DBN model well improves the final model. Even if the RMSE of sugar concentration resulting from the FGRAPE model seems to be better than the DBN’s one (6.11g/l to be compare to 7g/l), the FGRAPE model predictions of the total acid concentrations along time are lower than the DBN model predictions and do not allow to include further, variables that could be not measured by the experts, for example anthocyanins. Moreover the extreme values are better evaluated and some important errors are avoided which ensures more robust predictions. Thus for the Ac predictions the errors above 1 g/L are reduced from 4% to 2%. Same results are reached for the sugar predictions. This is of crucial importance if we want to propose a robust decision support system able to accompany the decision even if climatic conditions encountered are not those capitalized in the data basis.

Our aim is the prediction of the dynamics of the whole system, including the sugar and the total acid evolution over time. With this in mind, the formalism of DBN presents a very relevant platform, a kind of unifying framework to integrate multi-sources/scales of heterogeneous knowledge. That means that the concept of DBNs will allow to add new dimensions of representation as for instance grapes sensory properties linked to biophysical dynamics. Nevertheless, a DBN used alone, would have not predicted with a good accuracy the whole system as regards to the data available. In this sense, the integration of FGRAPE inside DBN implementing FGRAPEDBN, thus improves the RMSE of the whole coupled system and is a relevant way to reduce the uncertainty by the way of an integration of qualitative expert knowledge.

Parameter learning in FGRAPEDBN, for a known network structure, performs in polynomial time. However Inference in a dynamic Bayesian networks (see Eqs 7 and 15) is NP-hard [14]. The computational complexity of FGRAPEDBN does not stem from our methodology but from the chosen formalism of representation namely DBNs. Moreover, this coupling approach allows to reduce the uncertainty on the system by knowledge integration.

## Conclusion and Future Works

We have presented a way to build a robust decision help tool for grape maturity prediction. The originality is to associate experts' statements to a base mathematics constituted by the data of maturity of grapes. A coupling of two mathematical formalisms, fuzzy logic and dynamic Bayesian networks, is proposed and ensures this knowledge integration. Based on this system, software has been proposed and was currently used on the spot during last experimentation campaign. Further studies will focus on the generalization properties of such an approach.
